# Improving Glucose Homeostasis after Parathyroidectomy for Normocalcemic Primary Hyperparathyroidism with Co-Existing Prediabetes

**DOI:** 10.3390/nu12113522

**Published:** 2020-11-16

**Authors:** Spyridon Karras, Cedric Annweiler, Dimitris Kiortsis, Ioannis Koutelidakis, Kalliopi Kotsa

**Affiliations:** 1 Division of Endocrinology and Metabolism, First Department of Internal Medicine, Medical School, Aristotle University of Thessaloniki, AHEPA University Hospital, 54621 Thessaloniki, Greece; kalm@yahoo.gr; 2 Division of Geriatric Medicine, Department of Neuroscience, Angers University Hospital, 49035 Angers, France; ce.annweiler@chu.angers.fr; 3 Robarts Research Institute, Department of Medical Biophysics, Schulich School of Medicine and Dentistry, The University of Western Ontario, London, ON N6A 3K7, Canada; 4 Department of Nuclear Medicine, University of Ioannina, 45110 Ioannina, Greece; dkiorts@uoi.gr; 5 Second Department of Surgery, Gennimatas General Hospital, Aristotle University of Thessaloniki, 54124 Thessaloniki, Greece; Iokoutel@auth.gr

**Keywords:** normocalcemic primary hyperparathyroidism, parathyroidectomy, prediabetes, fasting glucose

## Abstract

We have previously described increased fasting plasma glucose levels in patients with normocalcemic primary hyperparathyroidism (NPHPT) and co-existing prediabetes, compared to prediabetes per se. This study evaluated the effect of parathyroidectomy (PTx) (Group A), versus conservative follow-up (Group B), in a small cohort of patients with co-existing NPHPT and prediabetes. Sixteen patients were categorized in each group. Glycemic parameters (levels of fasting glucose (fGlu), glycosylated hemoglobin (HbA1c), and fasting insulin (fIns)), the homeostasis model assessment for estimating insulin secretion (HOMA-B) and resistance (HOMA-IR), and a 75-g oral glucose tolerance test were evaluated at baseline and after 32 weeks for both groups. Measurements at baseline were not significantly different between Groups A and B, respectively: fGlu (119.4 ± 2.8 vs. 118.2 ± 1.8 mg/dL, *p* = 0.451), HbA_1c_ (5.84 ± 0.3 %vs. 5.86 ± 0.4%, *p* = 0.411), HOMA-IR (3.1 ± 1.2 vs. 2.9 ± 0.2, *p* = 0.213), HOMA-B (112.9 ± 31.8 vs. 116.9 ± 21.0%, *p* = 0.312), fIns (11.0 ± 2.3 vs. 12.8 ± 1.4 μIU/mL, *p* = 0.731), and 2-h post-load glucose concentrations (163.2 ± 3.2 vs. 167.2 ± 3.2 mg/dL, *p* = 0.371). fGlu levels demonstrated a positive correlation with PTH concentrations for both groups (Group A, rho = 0.374, *p* = 0.005, and Group B, rho = 0.359, *p* = 0.008). At the end of follow-up, Group A demonstrated significant improvements after PTx compared to the baseline: fGlu ((119.4 ± 2.8 vs. 111.2 ± 1.9 mg/dL, *p* = 0.021) (−8.2 ± 0.6 mg/dL)), and 2-h post-load glucose concentrations ((163.2 ± 3.2 vs. 144.4 ± 3.2 mg/dL, *p* = 0.041), (−18.8 ± 0.3 mg/dL)). For Group B, results demonstrated non-significant differences: fGlu ((118.2 ± 1.8 vs. 117.6 ± 2.3 mg/dL, *p* = 0.031), (−0.6 ± 0.2 mg/dL)), and 2-h post-load glucose concentrations ((167.2 ± 2.7 vs. 176.2 ± 3.2 mg/dL, *p* = 0.781), (+9.0 ± 0.8 mg/dL)). We conclude that PTx for individuals with NPHPT and prediabetes may improve their glucose homeostasis when compared with conservative follow-up, after 8 months of follow-up.

## 1. Introduction

Primary hyperparathyroidism (PHPT) is biochemically confirmed by hypercalcemia and inappropriately increased concentrations of parathyroid hormone (PTH) [1]. Apart from its well-documented musculoskeletal effects, PHPT has been associated with an increased prevalence of metabolic clinical conditions including disorders of glucose homeostasis [2,3,4,5]. However, thus far the potential metabolic benefits of parathyroidectomy (PTx) have not been established in these clinical conditions [3,4,5,6,7,8].

In vivo studies demonstrated that PTH administration has been associated with a reduction in insulin-stimulated glucose uptake, and a decrease in glucose transporter-4 and insulin receptor substrate-1 protein expression [2,3,4]. PTH also has been reported to down-regulate insulin intracellular signaling, resulting in an increase in peripheral insulin resistance [4,5,6], and is also inversely correlated with insulin sensitivity [5,6,7]. Epidemiological studies have also indicated that chronic inappropriate PTH secretion is a predisposing factor for the impairment of glucose homeostasis [7,8].

Normocalcemic primary hyperparathyroidism (NPHPT) is recognized as a new subclinical entity in the field of parathyroid disorders, and is characterized by increased serum PTH concentrations where serum calcium levels are within normal values, after the exclusion of other causes of high PTH [9,10]. In the field of metabolic complications, available results are conflicting regarding the effects of NPHPT as a potential risk factor for the development of cardiovascular and metabolic complications, although it has been associated with increased fasting glucose levels in patients with type 2 diabetes [11,12,13,14].

We have also reported that vitamin D deficiency, in combination with increased PTH, is associated with higher fasting glucose profiles in elderly individuals with prediabetes [15] and patients with co-existing NPHPT and prediabetes, compared to individuals with prediabetes per se [16]. However, the effect of a surgical intervention, compared to conservative follow-up in these populations, remains obscure.

This study evaluated the effect of parathyroidectomy (PTx) (Group A), compared to conservative follow-up (Group B), in a small cohort of individuals with co-existing NPHPT and prediabetes.

## 2. Materials and Methods

### 2.1. Study Population

The inclusion period was from December 2016 to March 2020. A total of 32 patients with NPHPT and prediabetes were initially included. NPHPT was defined as elevated serum PTH concentration (>65 pg/mL) and normal corrected serum Ca concentration [1,10]. Corrected Ca and PTH concentrations were measured on at least two occasions during a 3–6-month period, to confirm the persistence of the hyperparathyroid state. Prediabetes was diagnosed using the American-Diabetes Association (ADA) criteria, either as impaired fasting-plasma glucose (IFG) (fasting glucose (fGlu): 101–125 mg/dL) or impaired glucose tolerance (IGT) (2-h plasma glucose in the 75-g oral glucose tolerance (OGTT): 140–199 mg/dL), or as HbA1c values between 5.7% (39 mmol/mol) and 6.4% (46 mmol/mol) [17]. Exclusion criteria for both groups were as follows: (a) patients with pre-existing diabetes; (b) patients on any medication that could affect glucose metabolism and PTH dynamics (e.g., loop diuretics, lithium, denosumab); (c) previous medical thyroidectomy/parathyroidectomy; (d) conditions affecting vitamin D metabolism, such as malabsorption syndromes and chronic renal failure (stage 3–5); (e) patients with a body mass index (BMI) of >35 kg/m^2^; (f) hypercalciuria (>4 mg/kg body weight/day); (g) patients with familial hypocalciuric hypercalcemia (detected by 24 h urine Ca excretion and calcium-creatinine clearance formula) [1,18].

All participants underwent a physical examination and anthropometric measurements including height, body weight (BW), body mass index (BMI), waist circumference (WC), body fat (BF) mass and percentage, and lean body mass (LBM). Height was measured to the nearest 0.1 cm (cm) with a Holtain wall stadiometer. Waist circumference (WC) was measured midway between the lowest rib and the iliac crest by using an anthropometric tape. BMI was calculated as the ratio of weight in kilograms divided by the height in the meters squared (kg/m2). Body fat (BF) mass and percentage, muscle mass (MM) and lean body mass (LBM) were measured using bioelectrical impedance analysis (BIA) (SC-330S, Tanita Corporation, Tokyo, Japan).

Three months prior to baseline evaluation, all participants were supplemented with a daily vitamin D regimen (ranging from 1.200 to 4.000 IU) according to their latest available 25-hydroxy-vitamin D (25(OH)D) concentrations. This was in order to achieve 25(OH)D concentrations ≥ 30 ng/mL, according to international criteria [1,9,10], to avoid the effects of vitamin D deficiency on PTH values [18]. All participants underwent methoxy isobutyl isonitrile (MIBI) scintigraphy and parathyroid gland ultrasonography prior to inclusion. Imaging findings from parathyroid scintigraphy and ultrasonography were positive for the existence of a single parathyroid adenoma in 23 participants, whereas in four participants, findings were negative in ultrasonography and positive in scintigraphy. Finally, in the five individuals where no evidence of a parathyroid lesion was found, a conservative follow-up was suggested. According to a recent re-evaluation of these cases, two of these patients were confirmed as being positive for an adenoma (MIBI scintigraphy).

PTx was suggested to 21 participants at baseline, according to internationally adopted criteria for the management of PHPT [1] or patient preference. A total of five participants to whom PTx was suggested preferred conservative follow-up. Conservative follow-up consisted of a regular (3 month) biochemical evaluation of calcium homeostasis (total calcium (Ca), phosphorus (P), albumin, PTH, 25-hydroxy-vitamin D (25(OH)D), and 24 h urine calcium) for the assessment of hypercalcemia or hypercalciuria. Subsequently, participants were included in two groups, A (*n* = 16) and B (*n* = 16), following PTx or conservative approach, respectively. Anthropometric evaluation was repeated 32 weeks after PTx or conservative approach, for both groups. For the entire study period, participants in both groups followed regular dietetic guidance focused on prediabetes management according ADA recommendations [17]. A similar isocaloric diet was implemented for both groups, aiming at maintaining initial body weight. Participants were contacted via telephone and/or e-mail twice during the intervention to confirm their adherence to diets and to resolve any potential issues.

During the entire study period, participants were advised to maintain a stable level of physical activity, namely 150 min per week of moderate-intensity aerobic exercise, according to the ADA recommendations [17]. We did not use a specific method (e.g., a wearable device) to monitor physical activity. However, participants were strongly advised to conform to the recommendations, and the importance of such compliance was repeatedly emphasized by the research team. For the majority of participants, the suggested level of physical activity was slightly more intense than their habitual exercise status.

All participants were supplemented with a daily vitamin D regimen (1200 to 4000 IU daily) until the end of the study, with target 25(OH)D levels of ≥30 ng/mL [18]. Written informed consent was obtained from all participants. The study was conducted according to the Declaration of Helsinki for research involving humans. An ethical approval by the scientific committee was obtained by the Institution involved (Cyan Cross Hospital Ethics Committee).

### 2.2. Laboratory Evaluation

Laboratory evaluation was conducted both at baseline and after 32 weeks after PTx or conservative approach, for both groups. Blood samples were drawn after a 12-h overnight fast and stored at −20 °C prior to analysis. Fasting samples for total Ca, P, albumin, PTH, 25(OH)D, and glycemic parameters (fGlu, HbA1c, fasting insulin (fIns)) were obtained. Homeostasis model assessment (HOMA) was used for estimating insulin secretion (HOMA-B) and resistance (HOMA-IR) according to previously reported formulas [19]. Subsequently, all participants from both groups underwent a 75-g OGTT to evaluate 2-h post-load glucose response. Glucose and insulin measurements were performed using the Cobas INTEGRA clinical chemistry system (D-68298; Roche^®^ Diagnostics, Mannheim, Germany). Reference ranges were reported previously [16]. Corrected Ca was calculated according to the type Ca (mg/dL) + 0.8X (4 (mg/dL)—albumin (mg/dL)). Vitamin D status was assessed through the measurement of serum 25(OH)D, by competitive electrochemiluminescent immunoassay (Roche^®^ Modular E170). PTH was measured using the electrochemiluminescence immunoassay ECLIA (Roche^®^ Diagnostics GmbA, Mannheim, Germany). Coefficient variations and reference ranges have been reported previously for both assays [16].

### 2.3. Statistical Analysis

Continuous data are presented as mean ± standard deviation (SD). Comparisons among groups were performed using a Student’s *t*-test for unpaired data or using a Mann–Whitney U test. Proportions were compared with a Fisher exact test. The Pearson correlation coefficient (Pearson’s r) was also used for the examination of correlations of normally distributed variables, and Spearman’s rank correlation coefficient (rho) for non-normally distributed variables. A *p*-value < 0.05 was considered statistically significant. Two-way ANOVA analysis was conducted for comparisons between groups and within the same group.

Statistical analysis was performed using SPSS 13.0 software. We used the best available data of a suggested background prevalence of NPHPT in women of reproductive age, of 0.06% [12].

The required sample size was calculated using the STATA corporation statistical platform and was based on detecting a change in prevalence from 0.06% in the general population to 1% or more in our population. One per cent prevalence for NPHPT was chosen, as it is generally regarded as the lowest value that may stimulate a change in clinical practice. A one-tailed test with a power of 80% (i.e., *β* = 0.2) and significance level (*α*) of 5% showed a minimum requirement of 22 NPHPT patients with binomial 95% confidence intervals around the 1% prevalence of 0.23–3.19%. Descriptive statistics were calculated in Excel^®^ (V14.6, 2010, Microsoft^®^, Washington, USA).

## 3. Results

### 3.1. Baseline

Demographic, anthropometric, and biochemical data are presented in Table 1. Individuals in both groups did not differ with respect to age, female to male ratio, BMI, waist circumference, body fat, and lean body mass.

Both groups manifested a similar profile for calciotropic hormones (Table 1).

With respect to glucose homeostasis markers at baseline, fGlu (119.4 ± 2.8 vs. 118.2 ± 1.8 mg/dL, *p* = 0.451), HbA_1c_ (5.84 ± 0.0 vs. 5.86 ± 0.0%, *p* = 0.415), HOMA-IR (3.1 ± 1.2 vs. 2.9 ± 0.2, *p* = 0.211), HOMA-B (112.9 ± 31.8 vs. 116.9 ± 21.0%, *p* = 0.314), fIns (11.0 ± 2.3 vs. 12.8 ± 1.4 μIU/mL, *p* = 0.731), and 2-h post-load glucose concentrations (163.2 ± 3.2 vs. 167.2 ± 3.2 mg/dL, *p* = 0.371), were not different in the two groups at baseline (Table 2).

When calciotropic hormones were evaluated for interactions with glucose homeostasis, fGlu was positively associated with PTH concentrations (Group A, rho = 0.374, *p* = 0.005 and Group B, rho = 0.359, *p* = 0.008).

### 3.2. Following PTx or Conservative Approach

Anthropometric and biochemical features of calcium homeostasis for both groups are presented in Table 2. Individuals in both groups did not differ with respect to BMI (28.4 ± 0.6 vs. 28.8 ± 1.9 kg/m^2^, *p* = 0.652) and waist circumference (96.4 ± 1.2 vs. 97.1 ± 3.1 cm, *p* = 0.541).

At the end of follow-up, Group A demonstrated significant improvements in fGlu after PTx compared to baseline ((119.4 ± 2.8 vs. 111.2 ± 1.9 mg/dL, *p* = 0.021), (−8.2 ± 0.6 mg/dL)), as well as in 2-h post-load glucose concentrations ((163.2 ± 3.2 vs. 144.4 ± 3.2 mg/dL, *p* = 0.041), (−18.8 ± 0.3 mg/dL)). Group B demonstrated non-significant differences in fGlu ((118.2 ± 1.8 vs. 117.6 ± 2.3 mg/dL, *p* = 0.031), (−0.6 ± 0.2 mg/dL)) and 2-h post-load glucose concentrations ((167.2 ± 2.7 vs. 176.2 ± 3.2 mg/dL, *p* = 0.781), (+9.0 ± 0.8 mg/dL)) (Table 2).

As expected, calciotropic homeostasis was significantly improved in Group A compared to Group B, for PTH (44.2 ± 1.4 vs. 86.2 ± 2.2 pg/mL, *p* < 0.01), corrected Ca (9.1 ± 0.0 vs. 9.7 ± 0.2 mg/dL, *p* = 0.044), and P (3.9 ± 0.1 vs. 3.6 ± 0.1 mg/dL, *p* = 0.031), whereas 25(OH)D serum concentrations were similar for the two groups (32.3 ± 3.1 vs. 31.2 ± 1.9 ng/mL, *p* = 0.383) (Table 2). In addition, Group A demonstrated significant improvements vs. Group B in fGlu levels ((111.2 ± 1.9 vs. 117.6 ± 2.3 mg/dL, *p* = 0.02), (−6.4 ± 0.7 mg/dL)) and 2-h post-load glucose concentrations ((144.2 ± 3.2 vs. 176.2± 3.2 mg/dL, *p* < 0.01), (−32 ± 0.4 mg/dL)), respectively (Table 2).

## 4. Discussion

The purpose of this study was to investigate potential effects of PTx on glucose profiles of patients with co-existing prediabetes and NPHPT, versus conservative treatment. Our results indicated an improvement of glucose homeostasis following PTx, compared to the non-interventional strategy of conservative follow-up. This is the first report identifying an early (32 weeks post-surgery) beneficial glycemic effect of a surgical intervention (PTx) where there is the co-existence of both subclinical entities. The most plausible explanation for this early effect could result from the robust reduction of PTH concentrations after PTx, possibly explaining the improvement of glycemic parameters observed in our analysis.

Previous in-vitro studies indicated that increased concentrations of PTH have an adverse effect on mitochondrial oxidative phosphorylation, as well as adenosine triphosphate (ATP) islet content [20]. The increased milieu of calcium in the cytosol, as a result of the reduction of Ca exocytosis, has been associated with deterioration of glucose-stimulated insulin secretion, particularly in the preclinical or prediabetic state [21].

Of major interest are in vivo animal models which have indicated that PTH administration promotes a plethora of biological phenomena, including a decrease in insulin-stimulated glucose uptake, protein kinase B (AKT) activity (phosphorylated AKT/total AKT protein expression), and a reduction in glucose transporter-4 and insulin receptor substrate-1 protein expression. PTH also induced an increase of insulin receptor substrate-1 (IRS-1) phosphorylation on serine 307, which has been reported to down-regulate insulin intracellular signaling, resulting in an increase in peripheral insulin resistance. These results indicate that chronic PTH over-secretion is implicated in the development of dyshomeostasis on β-cells function, as well as development of insulin resistance in adipose tissue [20]. However, previous clinical human studies have not consistently reported a deterioration of peripheral insulin action [8,9,10]. In this study, no differences in HOMA-IR or HOMA-B indices between groups were evident, probably as a result of the small sample size.

In this regard, PTH has also been shown to inversely correlate with insulin sensitivity index (ISI), and has also been proven to be an independent negative determinant of ISI, with a decrease of 0.247 µmol/L/m^2^/min/pmol/L in ISI for each pg/mL increase in plasma PTH levels [22]. These reports were also confirmed by large-scale observational and epidemiological data, implying that elevated PTH concentrations are positively associated with abnormal glucose metabolism [23,24]. However, these results have not been confirmed by available intervention trials. We recently demonstrated a significant increase of glucose-stimulated glucagon-like peptide 1 secretion after successful PTx for asymptomatic primary hyperparathyroidism [25], indicating a favorable profile in incretion secretion physiology after surgical intervention.

Hagström et al. [12] evaluated the incidence of metabolic diseases (including diabetes mellitus) in 30 subjects with NPHPT (treated either with PTx or conservatively) during a five-year follow-up period, compared with age-matched controls. In their study [12], PTx had no effect on fGlu and HbA1c values. In this study, we also did not observe differences in HbA1c, probably due to the shorter follow-up period, which could outline a potential improvement in this setting. On the other hand, the discrepancy in fGlu concentrations was likely the result of differences in dietary practices and physical exercise between the two studies. In our study, a detailed dietary and exercise regimen was suggested to the participants, whereas in the Hagström et al. [12] study no such supervision was reported. Recently, a case–control study investigating the effect of PTx on cardiovascular risk factors in patients with normocalcemic and hypercalcemic PHPT [26] showed that after PTx improvements in blood pressure, serum total cholesterol, HOMA-IR, and cardiovascular risk scores were reported for both groups.

Our results suggest that in patients with NPHPT the reduction in PTH secretion after PTx results in significant improvement in the context of prediabetes only 32 weeks after surgery, indicating that surgical management of NPHPT might be a rational approach, particularly in cases where a deterioration of glucose homeostasis is evident during conservative follow-up. Although available guidelines [1,2,18] recommend that PTx is suggested primarily for musculoskeletal complications, our results indicate that PTx for metabolic reasons may be a future option.

A relative strength of our study is that we used strict criteria for diagnosing NPHPT and prediabetes, prior to patient inclusion in this analysis [18]. We have also excluded patients with other causes of increased PTH concentrations.

Main limitations include the lack of inclusion of a control group of participants with normal glucose homeostasis and other components of the metabolic syndrome (to draw more complete conclusions about the association between insulin resistance and dysfunction of parathyroid glands), the small sample size, and the use of HOMA indexes to evaluate insulin resistance and beta-cell function, instead of using the gold standard of clamping [27]. Moreover, we were not able to incorporate additional markers for evaluating insulin resistance and insulin secretion, such as the Matsuda index and the insulin secretion-sensitivity index-2. Finally, the follow-up period after PTx was relatively short in order to identify additional beneficial effects on glycemic homeostasis.

Measurements of additional components of the metabolic syndrome were absent, limiting the generalizability of these findings regarding the effects of PTx in the cardiometabolic profile of this cohort, as well as the association of insulin resistance with NPHPT. In this setting, more appropriately designed future studies could improve and elucidate the pathophysiological interplay between these preclinical entities.

In conclusion, these results indicate that PTx in individuals with NPHPT and prediabetes may improve glucose homeostasis compared to conservative follow-up, after 8 months of follow-up. Similar studies are required in larger population groups to confirm these results in the co-existence of these subclinical entities.

## Figures and Tables

**Table 1:** Comparative baseline demographic and anthropometric characteristics between groups.

Parameter	Group A	Group B	*p*-Value *
Participants; Women (*n* (%))	16; 12 (75%)	16; 11 (68.75%)	0.091
Age (years)	58.9 ± 1.0	56.2 ± 3.2	0.391
Weight (kg)	77.2 ± 18.8	77.6 ± 17.1	0.420
BMI (kg/m^2^)	28.1 ± 0.7	28.2 ± 1.3	0.814
Waist circumference (cm)	94± 1.9	96.1 ± 3.7	0.543
Body fat (%)	33.6 ± 7.6	35.4 ± 9.1	0.126
Lean body mass (kg)	50.7 ± 12.1	47.5 ± 9.9	0.283

Data are presented as mean ± standard deviation. *: Mann–Whitney test. Abbreviations: PTH: parathyroid hormone; BMI: body mass index; WC: waist circumference; 25(OH)D: 25-hydroxy-vitamin D.

**Table 2:** Comparative anthropometric and biochemical data throughout the study.

	Baseline	Week 32	*p*-Value for Trend within Groups *	*p*-Value for Group × Time Interaction *
*Weight (kg)*				
Group A	77.2 ± 18.8	77.8 ± 18.1	0.714	0.420
Group B	77.6 ± 17.1	78.0 ± 16.8		
*BMI (kg/m^2^)*				
Group A	28.1 ± 0.7	28.4 ± 0.6	0.811	0.652
Group B	28.2 ± 1.3	28.8 ± 1.9		
*Waist circumference (cm)*				
Group A	94.0± 1.9	96.4 ± 1.2	0.514	0.541
Group B	96.1 ± 3.7	97.1 ± 3.1		
*Body fat (%)*				
Group A	33.6 ± 7.6	34.7 ± 14.8	0.651	0.134
Group B	35.4 ± 9.1	32.2 ± 7.3		
*Lean body mass (kg)*				
Group A	50.7 ± 12.1	49.1 ± 15.6	0.783	0.178
Group B	47.5 ± 9.9	48.5 ± 10.4		
*PTH (pg/mL)*				
Group A	94.2 ± 2.4	44.2 ± 1.4 ^†,a^	< 0.01	< 0.01
Group B	96.2 ± 3.2	86.2 ± 2.2		
*25-hydroxy-vitamin D (ng/mL)*				
Group A	36.3 ± 2.1	32.3 ± 3.1	0.145	0.383
Group B	33.2 ± 1.3	31.2 ± 1.9		
*Serum corrected calcium (mg/dL)*				
Group A	9.9 ± 0.0	9.1 ± 0.0 ^†,a^	0.031	0.045
Group B	9.8 ± 0.1	9.7 ± 0.2		
*Serum phosphorus (mg/dL)*				
Group A	3.5 ± 0.0	3.9 ± 0.1 ^†,a^	0.011	0.031
Group B	3.4 ± 0.1	3.6 ± 0.1		
*Fasting glucose (mg/dL)*				
Group A	119.4 ± 2.8	111.2 ± 1.9 ^†,a^	0.021	0.020
Group B	118.2 ± 1.8	117.6 ± 2.3		
*Fasting insulin (μIU/mL)*				
Group A	11.0 ± 2.3	10.8 ± 1.1	0.601	0.731
Group B	12.8 ± 1.4	13.1 ± 1.8		
*HOMA-IR*				
Group A	3.1 ± 1.2	3.0 ± 1.1	0.631	0.213
Group B	2.9 ± 0.2	3.4 ± 1.1		
*HOMA-Β (%)*				
Group A	112.9 ± 31.8	114.1 ± 11.0	0.619	0.312
Group B	116.9 ± 21.0	114.2 ± 19.0		
*HbA1c (%)*				
Group A	5.84 ± 0.0	5.81 ± 0.0	0.411	0.511
Group B	5.86 ± 0.0	5.88 ± 0.0		
*2-h post-load glucose (mg/dL)*				
Group A	163.2 ± 3.2	144.4 ± 3.2 ^†,a^	0.041	< 0.010
Group B	167.2 ± 2.7	176.2 ± 3.2		

Abbreviations: PTH: parathyroid hormone; BMI: body mass index; WC: waist circumference; 25(OH)D: 25-hydroxy-vitamin D; HbA1c: glycated hemoglobin A1c; HOMA-IR: homeostatic model assessment for insulin resistance; HOMA-Β: homeostatic model assessment for beta-cell function. Data are presented as mean ± standard deviation. *: 2-way analysis of variance (ANOVA). ^a^: compared to baseline (comparisons within the same group). ^†^: compared to B group (comparisons between groups at the same time point).
